# The All Together Group: Co‐Designing a Toolkit of Approaches and Resources for End‐of‐Life Care Planning With People With Intellectual Disabilities in Social Care Settings

**DOI:** 10.1111/hex.14174

**Published:** 2024-08-08

**Authors:** Andrea Bruun, Amanda Cresswell, David Jeffrey, Leon Jordan, Richard Keagan‐Bull, Jo Giles, Sarah Swindells, Meg Wilding, Nicola Payne, Sarah L. Gibson, Rebecca Anderson‐Kittow, Irene Tuffrey‐Wijne

**Affiliations:** ^1^ Department of Public Health, Children's, Learning Disability and Mental Health, School of Nursing, Allied and Public Health Kingston University London Kingston Upon Thames UK; ^2^ Dimensions Reading UK; ^3^ MacIntyre Milton Keynes UK

**Keywords:** co‐production, death and dying, end‐of‐life care planning, experience‐based co‐design, intellectual disability, qualitative research, social care

## Abstract

**Introduction:**

Support staff within social care settings have expressed a need for resources to facilitate end‐of‐life care planning with people with intellectual disabilities. This study aimed to co‐design a preliminary toolkit of end‐of‐life care planning approaches and resources that can be implemented in adult social care services for people with intellectual disabilities.

**Methods:**

An adapted Experience‐Based Co‐Design process was applied to develop a toolkit for end‐of‐life care planning with people with intellectual disabilities. A co‐design group (the ‘All Together Group’) met six times from January to October 2023. The group comprised nine people with intellectual disabilities (including four researchers with intellectual disabilities, who also co‐facilitated the workshops), five family members, five intellectual disability support staff, two intellectual disability service managers, and five healthcare professionals.

**Results:**

The All Together Group tested resources for and approaches to end‐of‐life care planning with people with intellectual disabilities, based on findings from a scoping review and a focus group study. Easy‐read end‐of‐life care planning forms were deemed overwhelming and complicated, whilst visual and creative approaches were welcomed. Three new visual resources to support illness planning and funeral planning with people with intellectual disabilities were developed: (i) ‘When I'm ill’ thinking cards; (ii) ‘Let's Talk About Funerals’ conversation‐starter pictures; and (iii) ‘My funeral’ planning cards. These three resources, alongside three positively evaluated existing resources, were included in a new toolkit for end‐of‐life care planning with people with intellectual disabilities.

**Conclusion:**

Through an iterative, flexible, inclusive, and comprehensive co‐design process, a toolkit of three newly developed and three existing resources was created to facilitate support staff in doing end‐of‐life care planning with people with intellectual disabilities. Following a trialling process with support staff, the final toolkit was made freely available online.

**Patient or Public Contribution:**

The research team included four researchers with intellectual disabilities (A.C., D.J., L.J., and R.K.‐B). Researchers with intellectual disability have been part of every step of the research process; from study design to data collection and analysis to dissemination of study findings.Intellectual disability service provider representatives (M.W., N.P., and S.S.) were part of the co‐design group as well. Two of these representatives were also co‐applicants in the overall project (N.P. and S.S.). The co‐design group included people with intellectual disabilities, families, intellectual disability support staff and health and social care professionals. The study was supported by a Research Advisory Group comprising a variety of stakeholders, including people with intellectual disabilities families, intellectual disability researchers, representatives from intellectual disability organisations, and policymakers.

## Practitioner Points

- To facilitate proper and timely end‐of‐life care planning, resources and approaches should be developed together with people with intellectual disabilities and support staff to make sure they are relevant, accessible, and workable in social care practice.
- People with intellectual disabilities prefer visual and creative approaches to end‐of‐life care planning rather than easy‐read plans and forms.
- Co‐producing an end‐of‐life care planning toolkit is an iterative, time‐consuming, and complex process, which requires a flexible approach, adapting to the feedback from and needs of co‐design group members.

## 1 Introduction

Reviews and inquiries recommend that social care services engage in end‐of‐life care planning, involving people with intellectual disabilities and families [1, 2]. However, communication difficulties, issues with capacity, and death avoidance culture often prevent this [3, 4, 5]. There is also no guidance on how to involve people with intellectual disabilities in end‐of‐life care planning [6].

A scoping review and online survey identified a range of resources and approaches available for end‐of‐life care planning with people with intellectual disabilities [7]. These included resources specifically aimed at supporting people with intellectual disabilities to be involved in end‐of‐life care planning such as easy‐read advance care plans, where some had been (co‐)produced by intellectual disability service providers. Other resources included official guidance documents listing principles and values for end‐of‐life care planning, and resources aimed at supporting professionals in the planning process, for example, through online training. A recent focus group study found that support staff lack the skills, knowledge and confidence to do end‐of‐life care planning with people with intellectual disabilities [8]. It was highlighted that more support around communication was needed, particularly around how to discuss death and dying and how to initiate the conversation. An interview study with people with intellectual disabilities and their support staff showed that resources were helpful for staff when having end‐of‐life care planning discussions, where resources could help with structuring the conversation, approaching topics, and encouraging and prompting rich and detailed discussion [9]. It seems that despite a range of resources being available, social care support staff are not accessing or using them. An easily available toolkit of usable resources and approaches could help staff in supporting end‐of‐life care planning with people with intellectual disabilities.

An end‐of‐life care planning toolkit is more likely to be effective and workable if it is developed with the stakeholders it concerns. Thus, a co‐design approach is needed to properly include and consider those who are the toolkit end users. Evidence suggests that materials are perceived to be more applicable and acceptable to end users as a result of co‐design [10]. The central idea in co‐design is that service users provide the vital ingredients which allow public service professionals to be effective [11]. People with intellectual disabilities have also expressed how they want to have a say in what happens in services [12]. There is a growing body of co‐design research with people with intellectual disabilities within health and social care, including topics such as promoting physical health, social inclusion, housing, and mental health [13]. Guidelines on how to design and conduct inclusive health research with people with intellectual disabilities have been established by experts within the field as well [14]. Co‐designed research with people with intellectual disabilities has also been conducted on end‐of‐life care planning in New Zealand [15] and the Netherlands [16]. However, no such studies have been conducted in a UK context.

### 1.1 Previous Phases of the Project

This study was part of a wider project that aimed to identify principles, approaches, and shared decision‐making tools for end‐of‐life care planning that were most likely to be welcomed by people with intellectual disabilities, families, and staff in adult intellectual disability services, and to co‐produce end‐of‐life care planning guidance and resources for social care providers.

The first stage of the project involved a scoping review and online survey of the resources and approaches available for end‐of‐life care planning with people with intellectual disabilities [7].

The second stage was a focus group study with people with intellectual disabilities, families, support staff, healthcare professionals, and policymakers on their experiences and preferences for end‐of‐life care planning with people with intellectual disabilities [8].

This paper reports on the third stage of the project that involved co‐design of an end‐of‐life care planning toolkit for intellectual disability support staff.

## 2 Aim

To co‐design a preliminary toolkit of end‐of‐life care planning approaches and resources that can be implemented in adult social care services for people with intellectual disabilities.

## 3 Materials and Methods

The study design was an adaptation of Experience‐Based Co‐Design, as described by the Point of Care Foundation [17]. Experience‐Based Co‐Design aims to make meaningful changes to services by centring service users' experiences and collaborating with them on developing solutions to issues they raise. It involves making a ‘trigger film’ representing stakeholder views, which is used as a starting point to identify priorities and working together to co‐design service changes. The approach has gained increased interest within the healthcare sector [18], and it has been deemed promising in the development of palliative and end‐of‐life care services [19].

This adapted Experience‐Based Co‐Design study had two key elements:

1. Recordings from the focus group study were used to create a trigger film to highlight the views and experiences of different stakeholders. The focus groups explored the wishes and preferences of people with intellectual disabilities, families, and staff regarding end‐of‐life care planning [8].
2. Co‐design group workshops that assessed and critically appraised existing end‐of‐life care planning resources and approaches identified in the scoping review [7], and developed a testable end‐of‐life care planning toolkit. The trigger film created from the focus groups was shown in this group as a starting point for the work.

### 3.1 Sample and Recruitment

A co‐design group (renamed the ‘All Together Group’ by researchers with intellectual disabilities to be more accessible) with 20 members was formed.

Prospective group members were directly invited to take part in the study; some had taken part in the focus group study [8] or had been part of the Research Advisory Group. Prospective group members had to be available for all six sessions to take part in the study. Invitations were sent via email, including an easy‐read version for people with intellectual disabilities.

Group members read and agreed to an easy‐read ‘Terms of Reference’ document. This agreement was further discussed and revised in the first workshop.

The All Together Group comprised nine people with intellectual disabilities (incl. four researchers with intellectual disabilities), five family members, five support staff, two service managers, and five healthcare professionals. Three of the project's intellectual disability organisational leads (M.W., N.P., and S.S.) took part in the group as well as members of the research team. One Support Worker was also part of the group to support one of the group members with intellectual disabilities.

### 3.2 Research Team

The research team comprised a Professor (I.T.‐W.) with 20+ years' experience in end‐of‐life research involving people with intellectual disabilities; three Research Associates (A.B., R.A.‐K., and S.G.) with experience in co‐producing qualitative research, including within palliative care; four Research Assistants with intellectual disabilities (A.C., D.J., L.J., and R.K.‐B.) [20] with some research training [21] and end‐of‐life research experience; and a Research Assistant (J.G.), with end‐of‐life experience, supporting them.

### 3.3 Study Procedure

#### 3.3.1 Trigger Film

The trigger film is a key step of the Experience‐Based Co‐Design process as it is used as a starting point to identify priorities and work together to drive changes. A trigger film (length: 00:36:17 h) was developed based on focus group recordings. The film focused on key findings and issues arising in the focus groups and comprised short clips of focus group participants sharing their views. It was separated into two parts:

- Part 1: ‘End‐of‐life care planning… should we do it?’.
- Part 2: Four areas of end‐of‐life care planning (i.e., life planning, illness planning, funeral planning, and talking about death and dying).
  ○ When should we start end‐of‐life care planning?
  ○ Who should help to plan?
  ○ How should we do end‐of‐life care planning?
  ○ Staff support and training.

More detailed information about the focus group findings is provided elsewhere [8].

#### 3.3.2 Co‐Design Events

To accommodate the needs of people with intellectual disabilities and allow for a distribution of different group tasks, it was decided to split the co‐design group into two sub‐groups: an in‐person ‘Monday group’ with people with intellectual disabilities and an online ‘Thursday group’ with the remaining group members.

Each group took part in six co‐design workshops between January and July 2023. The final celebratory event was held in October 2023 in person. An additional session was held with the Monday group to get further feedback on the toolkit development. An overview of the workshops and their objectives can be found in Table 1. The specific activities for each workshop can be seen in Appendix S1.

**Table 1:** Co‐design group workshops.

Month and year	Workshop no.	Workshop objectives
January 2023	Workshop 1	Present an overview of selected approaches and resources from the scoping review and trigger film.
February 2023	Workshop 2	Agree on key principles and preferred approaches for an end‐of‐life care planning toolkit.
March 2023	Workshop 3	Agree on core competencies for intellectual disability services providers and staff skills in implementing end‐of‐life care planning.
	Workshop 4	Agree on key elements of end‐of‐life care planning tools and resources and assess selected resources in light of these; select resources to include in the toolkit and/or guidance for tool development; identify what new/additional/adapted resources are needed.
April 2023	Workshop 5	Continuation of workshop 4; and make final decisions on the content and method of end‐of‐life care planning training.
May 2023	*Monday group additional session*	Try the No Barriers here approach to end‐of‐life care planning.^a^
June 2023	Workshop 6	Appraisal of the final toolkit and any final discussions.
October 2023	Celebration	

a The No Barriers Here workshop was cancelled due to facilitator illness.

The Monday group workshops with people with intellectual disabilities were held using accessible, inclusive and creative methods. This group had full‐day (5 h) in‐person sessions. These workshops were co‐facilitated by researchers with intellectual disabilities (A.C., D.J., L.J., and R.K.‐B.). An easy‐read agenda was sent out to group members before each meeting. Each meeting began with ice‐breaker games [22] and ended with a reflection round.

The Thursday group met online on Zoom for 2 h. A minimum of one researcher with an intellectual disability would participate in the meeting and feedback to the Thursday group about what the Monday group had done. The first part of the Thursday meeting was dedicated to sharing what the Monday group had worked on and what their views were. The research team made short videos of the Monday group activities to present key points.

Field notes were taken during and/or after each workshop by the research team. The research team discussed each workshop's ‘findings’ (i.e., feedback from and outcomes of group discussions and activities). In between workshops, the research team planned activities for the subsequent workshop, using the protocol as a guideline (see Appendix S1). Researchers with an intellectual disability trialled and approved activities and resources before each workshop with the Monday group, to ensure they were accessible and workable in practice. Based on workshop findings, prototypes of resources were developed. This was a comprehensive iterative (feedback) process of listening to the groups, presenting new ideas or resources for them to try or discuss, getting group feedback on ideas and resources, and fine‐tuning and editing ideas and resources, which were then brought back to the groups for final or further feedback and discussion.

## 4 Results

Following the production of the trigger film and discussion with the All Together Group, the research team decided that the focus of toolkit development should be on resources that targeted the ‘funeral planning’ and ‘illness planning’ aspects of end‐of‐life care planning. Life planning is an important area that feeds into planning for the end of life, but this should be part of the person's support plan already in place within intellectual disability services. A body of research has focused on how to talk about death and breaking bad news to people with intellectual disabilities [23, 24, 25, 26, 27, 28]. Creating a culture where staff are able to talk about death and dying is important, but enabling this to happen within all services requires a culture and organisational change. Even within the wider society, death is seen as a taboo and not spoken about, and there are significant challenges with changing the culture [29]. For these reasons, it was deemed beyond the remit of the project to focus on talking about death and dying. It was decided to focus on resources to support end‐of‐life conversations, with some guidance for staff on how to talk about dying.

### 4.1 Testing Existing Resources

The Monday group with people with intellectual disabilities tried out accessible resources that had been identified in the scoping review. Most of these resources were easy‐read end‐of‐life care plans. Other resources included Talking Mats [30] and the No Barriers Here approach [31].

#### 4.1.1 Easy‐Read End‐of‐Life Care Planning Forms

The Monday group tried and assessed several easy‐read end‐of‐life care planning forms. Some of these were co‐produced with people with intellectual disabilities by intellectual disability service providers and involved plans for care at the end‐of‐life as well as funeral plans.

The group was overwhelmed with the number of pages in the forms. They expressed how many of the forms were not engaging, and that a lot of them involved complicated language (‘jargon’). Their stark negative feedback included not wanting to pick up the forms and even putting them in the bin. It became clear that easy‐read forms were not a welcomed approach by this group, and it was decided not to include any of the forms in the toolkit, nor develop any new easy‐read forms.

#### 4.1.2 Talking Mats

The Monday group tested Talking Mats, which is a visual communication framework that supports people with communication difficulties in expressing their feelings and views [30]. The (physical or digital) mat comprises three sets of picture communication symbols (i.e., topic, options, and a visual scale) and a space to display them.

The group tested the Talking Mats Thinking Ahead resource that covers care and treatment wishes, affairs, and personal values. They also tested a new Funeral Planning resource that had been released by Talking Mats for internal testing. Topics included in this resource are funeral planning, service planning, and the eulogy. The approach was welcomed by the group, and they expressed a preference for this approach as opposed to the easy‐read forms. They mentioned how it was more visual, quick, and straightforward, so it was decided to include it in the toolkit.

#### 4.1.3 No Barriers Here

No Barriers Here is an equity‐oriented, art‐based approach to advance care planning that is aimed at people who may be marginalised in healthcare [32]. It is delivered through three workshops that each explores a different aspect of advanced care planning, using different art‐based methods to enable verbal and less verbal exploration and expression of views, experiences, and preferences.

The Monday group was scheduled to try the No Barriers Here approach, but this session was cancelled due to facilitator illness. After the official last workshop, the group was again invited to try No Barriers Here, but none of the group members could make it. Only members of the research team, including researchers with and without intellectual disabilities, tried the approach. The approach was deemed inclusive and useful by all researchers and was included in the final toolkit.

#### 4.1.4 *Am I Going to Die?* Book Beyond Words

Beyond Words is a UK charity that co‐creates word‐free picture stories aimed at helping people understand and communicate their feelings, learn about new experiences, and tell their own stories [33]. *Am I Going to Die?* is one of their books, which is about a man with an intellectual disability who gets terminally ill and eventually dies from his illness.

As the focus group study showed that people with intellectual disabilities (as well as people in the general population) find it difficult to make illness plans in advance [8], the book was not tested by Monday group members. However, the book seemed promising in the focus group study, where it was found to aid discussions about terminal illness. It aligned with the visual approaches that the group had welcomed and was therefore included in the toolkit.

### 4.2 Development of New Resources

The initial testing of resources and observations showed that there was a lack of visual and creative approaches to end‐of‐life care planning with people with intellectual disabilities. Talking Mats, No Barriers Here, and *Am I Going to Die?* seemed promising. However, these resources are not freely available. Both Talking Mats and No Barriers Here require the facilitator to undergo training in the approach for a fee. They also involve acquiring and/or purchasing relevant materials and resources. Although less expensive and complex to acquire, the *Am I going to Die?* book involves purchasing a copy of the book. To address this gap in the available resources, it was decided to develop new freely available resources.

#### 4.2.1 Funeral Planning

Two resources for funeral planning were developed: *My funeral* cards and *Let's Talk About Funerals* pictures. *My funeral* cards are cards used for making specific choices about one's funeral. *Let's Talk About Funerals* is a set of pictures designed to start conversations about death, funerals, and funeral planning. An overview of the key steps of the development process of each resource is described below. A more detailed overview can be found in Appendix S1.

##### 4.2.1.1 My Funeral Planning Cards

The Talking Mats visual approach to funeral planning was clearly favoured over a form‐based approach. There was a need for a wider range of visual or creative resources; therefore, it was decided to develop an alternative way to support funeral planning.

The first attempt involved a ‘fold out’ funeral plan, consisting of four areas of funeral planning, where the person could fill in their wishes. The plan included sheets with pictures (e.g., different coffins, flowers, etc.) that the person could cut out and glue into the plan. The person could also find their own pictures, draw or simply write down their wishes. This way of doing funeral planning was trialled and welcomed by just one group member. The other group members found it confusing, multiple picture options were overwhelming, and they did not want to engage with it.

The failure of the initial funeral planning resource forced the research team to think differently about what the resource should do. It was decided to experiment with single‐image, single‐topic planning cards, which could be used to talk about the topic, and if the person wanted to, to express preferences or choices. This was inspired by co‐produced outputs of a previous study, which consisted of similar cards aiming at planning for the future with people with intellectual disabilities [34]. The initial stages of developing the resource involved finding funeral images online and testing how these worked in practice. Both groups welcomed the idea, and a list of images needed for the set of cards was then created. An artist was commissioned to draw the images for the cards.

The artist sent black‐and‐white drafts for the group to approve. Some pictures also had different options that the group could choose from. Second versions were produced following requests for changes by the research team, including a strong demand from group members that the pictures were in colour. Therefore, the research budget and timeline needed to be increased to ensure colour versions could be created. Colour versions were checked by the group and any changes requested were fed back to the artist. Drawings were completed after the last group meeting, and the final ones were checked and approved by the research team, including researchers with intellectual disabilities.

The final resource included 17 cards divided into four main sections:

1. What happens with my body (e.g., Burial and Cremation).
2. The day of my funeral (e.g., Music and Flowers).
3. Remember me (e.g., Celebration and My things).
4. Plan my funeral (i.e., Who helps me plan and Paying for my funeral).

There was a separate recording sheet to note the person's decisions.

##### 4.2.1.2 Let's Talk About Funerals Conversation‐Starter Pictures

As visual and creative approaches and the Beyond Words materials seemed promising, it was decided to test whether pictures were a useful way of initiating the conversation about funerals.

There is no Beyond Words book specifically dealing with funerals, going to a funeral or planning a funeral. For this reason, the research team scoped their books for any funeral‐related pictures. These pictures were isolated from their respective books and shown to the group. This was highly successful, leading to open and positive conversations about the pictures. The pictures prompted people with intellectual disabilities to share their own experiences of funerals and even their own funeral preferences. The first trialling round included a few questions with each picture, that could be used by a supporter to prompt the conversation. However, these were found to restrict the conversation and disturb the conversational flow. Based on these observations, it was decided to develop new conversation‐starter pictures in collaboration with Beyond Words, without any words or questions that might take the power and leadership of the conversation away from the person. The idea and approach were welcomed by the Thursday group as well.

The Monday group discussed which new pictures were needed to ensure that people would be able to talk about many different aspects of funerals. A list was created and a Beyond Words artist was commissioned to draw new pictures and to adapt existing ones. Black and white drawings were trialled with the group and changes were requested before developing the colour versions. The pictures were also trialled as part of a focus group with people with intellectual disabilities from minoritised ethnic groups [35]. The final resource included 14 pictures of different scenarios from different types of funerals.

#### 4.2.2 Illness Planning

The focus group study and All Together Group had one crucial limitation: none of the participants with intellectual disabilities were terminally ill or approaching the end‐of‐life. The Monday group tested easy‐read end‐of‐life care plans and Talking Mats. However, as all the group members with intellectual disabilities were fit and well, their responses to what choices they would make in their final months of life were rather vague. It became increasingly clear that specific choices for illness can be difficult to make in advance. Illness planning can be rather abstract and hypothetical, where specific end‐of‐life scenarios, choices or limitations depend on circumstances that can be hard to predict. It was mostly the Thursday group who worked on the illness planning aspect, as they found it easier to understand and imagine future options, as well as the limitations of the extent to which it is possible to plan ahead.

The research team decided to invite palliative care professionals from the Research Advisory Group and focus groups for an online chat about what Support Workers' role is in end‐of‐life care planning. These one‐to‐one chats with professionals revealed that their key role was to be the bridge between the healthcare service and the social care provider**—**not to make or plan specific care decisions (in advance).

Based on the additional discussions, it became clear that it was useful for the palliative care professionals to know the following about the person with intellectual disability that the Support Worker could help with:

○ What does a good day look like for the person? (What is their best day?)
○ What does a bad day look like for the person?
○ What are their past experiences of hospital visits/treatments?

Following this, the research team further developed resources that targeted this approach to illness planning. This took the form of another set of cards *When I'm ill* cards. An overview of the remaining key steps of the development process is described below. A more detailed overview can be found in Appendix S1.

##### 4.2.2.1 When I'm Ill Thinking Cards

The visual approach that the Monday group members had welcomed was continued. Thus, it was decided to create cards that could help the person with an intellectual disability to think in general about health‐related matters, and not cards focusing on making actual decisions.

One of the palliative care professionals from the initial chat about the role of support staff was asked to create a list of healthcare interventions that may be relevant to ask the person. This led to a list of images needed to create a similar set of cards as *My funeral* cards. The Thursday group welcomed this approach, and the same artist was commissioned to draw the images for the cards. A similar process as the one described for the *My funeral* cards was applied. This included creating black‐and‐white versions of the drawing for the group to approve, feedback to the artist, and subsequent development of colour versions. The Thursday group tried the black‐and‐white versions and found the approach and cards very useful. They also came up with suggestions for other cards.

The final resource included 26 cards divided into five main sections:

1. About me (e.g., Good day and Bad day).
2. Where (e.g., Hospital and Home).
3. Treatment (e.g., Taking medication and Needles).
4. Needing help (e.g., Help with walking and Being cared for in bed).
5. Before I die (i.e., What I want to do when I still can and Saying goodbye).

There was a separate recording sheet to note the person's thoughts about each card.

It was important to stress that these cards were not about *making decisions* but about thinking and exploring the topics together. For this reason, the research team created questions that the Support Worker could have in mind when using the cards. Taking the ‘Hospital’ card as an example, the questions would be:

- What would it be like for you to go into hospital?
- What is good about going into hospital?
- What is difficult about going into hospital?
- What might make it easier for you to go into hospital?

When the cards were trialled by the Monday group members, the research team was very mindful that they were not ill. Nonetheless, group members reported having positive discussions about some of the cards using these questions, for example, why going into hospitals can be difficult or what may help someone if they are in a situation where they must take certain kinds of medication.

An important role of the Thursday group was to represent and consider the views of people with severe and profound intellectual disabilities. Together with the group, research on how to involve people with severe and profound intellectual disabilities in end‐of‐life decision‐making [36, 37] was explored. This research highlights the importance of the relationship with and knowledge of the person with intellectual disabilities when having to make end‐of‐life care planning decisions. The key points resonated with the Thursday group, about involving several people in the planning process and how stories about the person and what they like can be used to inform their end‐of‐life care plans. The group found that the *When I'm ill* cards (and the *My funeral* cards) were helpful and workable with people with severe and profound intellectual disabilities, as families and support staff were able to speak about the different topics on the cards considering the wishes and preferences of the person they knew well.

#### 4.2.3 Finalising the Resources and Toolkit

After the last workshop, the Monday group was asked if they could be contacted if feedback on the drawings was needed before finalising them for the evaluation study. They were contacted via email to provide informal feedback on some of the pictures. They were also asked for feedback on the proposed titles of the resources. At the celebration event, the final pictures were presented, and each group member received a copy of the resources.

Four of the toolkit resources (i.e., *My funeral* cards, *Let's Talk About Funerals, When I'm Ill* cards, and *Am I going to die?*) were trialled in an evaluation study with intellectual disability support staff, who provided feedback on the toolkit. The evaluation study will be reported elsewhere. Staff feedback obtained in this study was used to decide if any new pictures or cards were needed. A list of additional cards and pictures was developed and commissioned. The Monday group was asked to review the new cards and pictures in an additional meeting online in March 2024. Their feedback was summarised to the artists who finalised the pictures. The final toolkit was launched at a conference in June 2024 and made available on the study website (www.victoriaandstuart.com).

## 5 Discussion

This study showed that people with intellectual disabilities preferred visual approaches to end‐of‐life care planning conversations. This study finding aligns with other findings from similar Experience‐Based Co‐Design research within the intellectual disability field [34] and projects working on creating awareness and increasing comfort in talking about death and dying with people with intellectual disabilities [38]. Visual approaches to decision‐making such as Talking Mats have been shown to be effective [39, 40]. Conversation‐starter resources for advanced care planning have been received positively when used with people with dementia [41] and with members of the general population [42, 43]. A meta‐analysis also showed that games for advance care planning were effective in increasing self‐efficacy, readiness and knowledge, and that games were deemed highly acceptable, fun, and enjoyable by participants [44]. This advocates for the general acceptability and feasibility of more alternative approaches to end‐of‐life care planning, moving away from papers or forms.

It is unsurprising that people with intellectual disabilities in the Monday group did not like lengthy easy‐read plans. Whilst some of these plans have been co‐produced with people with intellectual disabilities, forms comprising more than 20 pages were overwhelming for the group members. This is in line with research showing that the number of sentences and text length may hinder comprehension and that an increase in reading time is linked with people not liking easy‐read texts [45]. Too many pages of easy‐read have also been described as boring and stressful by people with intellectual disabilities [46].

The developed toolkit resources are primarily pictorial and visual and involve either no or a very limited number of words. Any words included in the resources have been carefully chosen and approved by the group with people with intellectual disabilities. This was to make sure they were clear and understandable. In this way, the resources align with communication recommendations by The European Association for Palliative Care stressing that written information should entail clear words and pictures to promote understanding [47]. Ensuring good communication with people with intellectual disabilities has also been deemed of high importance by The European Association for Palliative Care [47], and good communication has been described as a facilitator for palliative care provision with this population [3]. The toolkit resources identified and developed in this study aim to facilitate good communication about end‐of‐life care planning between support staff and people with intellectual disabilities.

### 5.1 Reflection on the Co‐Design Process

A flexible approach is needed to conduct a meaningful and non‐tokenistic inclusive research co‐design project. In this study, that meant that the initial plan of having six 2‐h Co‐design workshops for people with intellectual disabilities (also planned to be on the same day as the workshops with the other group) had to be revised. Two hours proved to be much too short, and longer workshops were needed. Having two workshops on the same day was also deemed infeasible as it did not allow for the research team to reflect on and work with the findings between workshops. Already having the workshops in the same week meant a significant workload was added to the research team when preparing the updates for the Thursday group.

Preparing accessible materials, including dissemination materials (such as videos), is highly labour‐intensive. In this study, the research team was also designing new resources which added to the work burden. Workshops with people with intellectual disabilities needed to be planned well in advance, to ensure they could prepare for the activities and organise sufficient support. Having monthly workshops over six months was indeed a challenge. The intense and fast pace of the process also meant that it was difficult to keep researchers with intellectual disabilities abreast of the process. A similar study described how a longer timeframe than six months was positively received and allowed time for reflection, integration of feedback, and flexibility [34].

As evident in the descriptions of the development process of the resources, the research team had to think outside the box, which further underlined the iterative nature of the project and the flexibility needed. Particularly, the development of the illness planning resources added to the complexity of the project, and complexity has indeed been described as inherent to Experience‐Based Co‐Design studies [18].

It was essential to have adequate support in place for the Monday group both during and in between workshops. Having researchers without intellectual disabilities, three intellectual disability organisational leads and a Support Worker as part of the group meant that group members with intellectual disability could be supported to understand the process in a way that made sense to them. JG also played an important role in supporting researchers with intellectual disabilities in workshop co‐facilitation.

An important element of the study was the relationships that were built throughout the co‐design process, particularly within the Monday group. Having ice‐breaker games and reflection rounds was important to create a safe space where group members with intellectual disabilities could speak openly. The balance that all group members created in the sense of valuing each other's inputs was also stressed in the Terms of Reference agreement, and the sharing of power was instrumental in creating a space where people could share their honest opinions. Being able to share thoughts and honest opinions was also crucial in terms of getting feedback when developing resources to ensure their feasibility and acceptability in practice.

Creating a safe space was also important when dealing with a tender topic such as end‐of‐life care planning, particularly as people with intellectual disabilities are often reported as being excluded or shielded from such discussions [48]. An essential part of creating a safe space was to have fun together. During every Monday group workshop, the group would dance, sing, and laugh. However, there was also space and support for people to be emotional and cry. This further highlights the trust that was built, which allowed people to not only express their opinions but also express their emotions.

The relationship‐building and safe space also meant that confidence grew within the groups. Some group members had been reluctant to discuss end‐of‐life care planning when they took part in the focus groups. Throughout the process, group members became more comfortable with discussing the topic. At the last picture feedback session, one Monday group member, who had been reluctant to even look at a funeral picture during the focus groups, shared how they had now paid and planned their own funeral. This stresses how talking about the topic can take time, requires trusting and good relationships, and a safe space.

### 5.2 Strengths, Limitations and Future Research

A key strength of this study is the toolkit development using a co‐design process with relevant stakeholders involved in end‐of‐life care planning with people with intellectual disabilities. The extended length and number of workshops with people with intellectual disabilities meant that the resources had been thoroughly co‐designed by key stakeholders. This increases the likelihood of the toolkit being relevant and workable in practice.

It was deemed necessary to split the co‐design group into two separate ones. Initially, 2‐h workshops for both groups were planned to be held on the same day; however, it became clear that the Monday group needed more time to work with things and these events were changed to full‐day in‐person sessions instead. To mitigate the split between groups, it was ensured that a significant amount of time in the Thursday group meetings was dedicated to presenting what the Monday group had done. In future studies, it should be explored whether such groups can be meaningfully and effectively combined.

This study did not include people with severe and profound intellectual disabilities. Their perspectives were represented by family members and support staff by proxy. Future research should explore the views of people with severe and profound intellectual disabilities further. Research is also needed to inform how people with severe and profound intellectual disabilities can be meaningfully included in co‐design studies.

## 6 Conclusion

Through a thorough and inclusive adapted Experience‐Based Co‐Design process, a toolkit to support end‐of‐life care planning with people with intellectual disabilities was developed. People with intellectual disabilities preferred visual and creative approaches and resources over easy‐read forms. For this reason, three new resources were developed: *Let's Talk About Funerals* conversation‐starter pictures; *My funeral* planning cards; and *When I'm ill* thinking cards. These resources were included in the toolkit together with Talking Mats, No Barriers Here, and the *Am I Going to Die?* book. By including people with intellectual disabilities, families, support staff, and healthcare professionals throughout the development process, resources have been created that reflect and meet the needs and preferences of support staff facilitating end‐of‐life care planning conversations and people with intellectual disabilities.

## Author Contributions

**Andrea Bruun:** conceptualisation, methodology, formal analysis, writing–original draft, writing–review and editing, project administration, data curation, investigation. **Amanda Cresswell:** conceptualisation, methodology, investigation, writing–review and editing, formal analysis. **David Jeffrey:** investigation, writing–review and editing, formal analysis, methodology. **Leon Jordan:** formal analysis, writing–review and editing, investigation, methodology. **Richard Keagan‐Bull:** conceptualisation, methodology, formal analysis, writing–review and editing, funding acquisition, investigation. **Jo Giles:** investigation, writing–review and editing, formal analysis, methodology, validation. **Sarah Swindells:** conceptualisation, funding acquisition, investigation, methodology, formal analysis, writing–review and editing, validation. **Meg Wilding:** investigation, writing–review and editing, formal analysis, methodology, validation. **Nicola Payne:** conceptualisation, methodology, investigation, funding acquisition, writing–review and editing, formal analysis, validation. **Sarah L. Gibson:** writing–review and editing, investigation, formal analysis, methodology, project administration. **Rebecca Anderson‐Kittow:** conceptualisation, methodology, writing–review and editing, funding acquisition, investigation. **Irene Tuffrey‐Wijne:** data curation, conceptualisation, methodology, writing–review and editing, project administration, supervision, formal analysis, funding acquisition, investigation.

## Ethics Statement

The study received ethical approval from the West Midlands**—**Coventry & Warwickshire Research Ethics Committee (22/WM0026) on 22/04/2022.

## Consent

Co‐design members were considered extended members of the research team (akin to PPI members), rather than research participants. They were therefore not asked to provide consent and were given terms of reference for the group rather than a participant information sheet. It was important to highlight equality across the co‐design team, rather than splitting it into researchers and research participants. The paper does not contain reproduced materials from other sources thus permission has not been obtained.

## Conflicts of Interest

Prof Irene Tuffrey‐Wijne is a Beyond Words trustee and one of the authors of the *Am I Going to Die?* book.

## Supporting information

Supporting information.

## Data Availability

The outcome of the study was an end‐of‐life care planning toolkit. The toolkit was revised based on feedback obtained from a follow‐up study testing the toolkit with intellectual disability support staff. The final toolkit and its resources are freely available online on the study website: www.victoriaandstuart.com. Other relevant and shareable study data are available in Appendix S1.
