# Supplementary material for: The All Together Group: Co‐Designing a Toolkit of Approaches and Resources for End‐of‐Life Care Planning With People With Intellectual Disabilities in Social Care Settings
Source: Health Expect. 2024 Aug 8;27(4):e14174. doi: 10.1111/hex.14174 (PMC11306969; doi:10.1111/hex.14174)
Supplement: Supplementary file 1 — Supporting information. [file HEX-27-e14174-s001.docx]

# Appendix 1: Workshop objectives and activities

| **Workshop #** | **Objective according to protocol** | **Activities Monday group** | **Activities Thursday group** |
| --- | --- | --- | --- |
| Workshop 1 | Consider and reflect on scoping review and focus group data. Present an overview of selected approaches/resources from the scoping review and the film of stakeholder views developed from the focus groups. This is scene-setting, so that the group gets to grips with end-of-life care planning concepts, principles, stakeholder preferences and possibilities. | - Ice-breaker games (with hats and soft toy) - Group ground rules. - Trigger film Part 1; broken into smaller parts with 15 minutes discussion for each aspect of end-of-life care planning (funeral planning, illness planning, life planning, and talking about dying). - Reflection round (with soft toy). | - Project and study presentation. - Introductions. - Group ground rules. - Trigger film and questions for discussion. - Scoping review results. |
| Workshop 2 | Agree key principles and preferred approaches for an end-of-life care planning toolkit. We will set out the core components of existing approaches, and select workable “who, when, how” components of the new programme | - Ice-breaker games (with hats and soft toy). - Group ground rules recap. - Washing line game: When should end-of-life care planning start by pinning picture onto a washing line representing the life of a terminally ill man (from baby to death). - Reviewing easy-read end-of-life care planning forms. - Trying Talking Mats (one group member). - Reflection round (with soft toy). | - Recap of last meeting. - Update from Monday All Together Group. - Google Jamboards to record thoughts on what is important about the four parts of end-of-life care planning. - Open discussion. |
| Workshop 3 | Agree core competencies for intellectual disability services providers and staff skills in implementing end-of-life care planning. This will build on 10 core competencies needed by intellectual disability staff involved in end-of-life care planning, identified by Voss et al. (2021) | - Ice-breaker games (with hats and soft toy). - Trying Talking Mats (three group members). - White Sheet Game (group members pinned their thoughts and ideas about what kind of support worker they wanted onto a sheet worn by a researcher with intellectual disabilities). - A personal washing line (a researcher with intellectual disabilities presented his life through pictures pegged onto a washing life) – people were given string and pegs if they wanted to do one themselves. - Reflection round (with soft toy). | - Recap of last meeting. - Update from Monday All Together Group. - Google Jamboards to record thoughts on what competencies support staff need to do the four parts of end-of-life care planning. |
| Workshop 4 | Agree key elements of end-of-life care planning tools and resources; assess resources selected in the joint scoping review and focus group study workshop in light of these; select resources to include in the toolkit for testing in the next stage of project and/or guidance for tool development; identify what new/additional/adapted resources are needed | - Ice-breaker games (with hats and soft toy). - Reflecting on the personal washing line from last session. - Discussion about Beyond Words and using pictures to talk about funerals. - Trying picture conversation cards (pictures of funerals with question prompts at the back of the cards). - Trying out a fold-out funeral plan developed by the researchers (included four areas of funeral planning, where the person could fill in their wishes). - Voting exercise about what people thought about Talking Mats, Beyond words, or something else. - Reflection round (with soft toy). | - Recap of last meeting. - Update from Monday All Together Group. - Thinking about choices for end-of-life with people with severe/profound intellectual disabilities (presentation of research within the area, including a video). - Breakout room discussions based on conversations with palliative care professionals about what may be useful questions to ask regarding illness planning, including using conversation cards. |
| Workshop 5 | Continuation of workshop 4, plus: Make final decisions on the content and method of end-of-life care planning training and implementation programme | - Ice-breaker games (with hats and soft toy). - Reflecting on the fold-out funeral plan from last session (one group member had filled it in and presented it to the group). - Drawing a cremation. - Trying the funeral choice cards (two group members) and think about cards that should be added to the deck. - Discussing the Beyond Words conversation pictures (i.e., what topics you may talk about with each image and what new images are needed). - Discussing easy-read forms and what they should/could be used for. - Reflection round (with soft toy). | - Recap of last meeting. - Update from Monday All Together Group. - Conversation cards for illness planning task (group members tried a card in a lightning round format in breakout rooms and feedback to the whole group afterwards). - Conversation cards for illness planning discussion (Are they useful? Which cards are needed?) |
| Additional workshop | Trying the No Barriers here approach to end-of-life care planning*. | - Ice-breaker games (with hats and soft toy). - Watching a video about the No Barriers Here approach to end-of-life care planning. - Looking at and talking about new Beyond Words pictures and suggesting changes. - Trying and discussing Illness cards pictures and suggesting changes. - Trying filling in easy-read funeral forms. - Reflection round (with soft toy). |  |
| Workshop 6 | Appraisal of the final toolkit and implementation programme, and any final discussions. | - Ice-breaker games (with hats and soft toy). - Looking at and talking about new funeral and illness planning pictures and suggesting changes. - Looking at and talking about new Beyond Words pictures and suggesting changes. - Video making for staff for the toolkit. - Reflection round (with soft toy). | - Recap of the five meetings. - Presentation of the preliminary resources for funeral planning and illness planning. - Reviewing the list of funeral cards and suggesting changes. - Reviewing the list of illness planning cards and suggesting changes. - Beyond Words pictures (discussing the new cremation picture). - Discussion of guidance for resources. - Staff training format discussion (for trialling resources in the next stage of the project). - Top tips for doing this funeral and illness planning topic (each group member shared their top tips). |
| *Celebration event* | | Games, quizzes, presenting final version of resources, and certificate ceremony. | |

*The No Barriers Here workshop got cancelled due to facilitator illness.
